# Prevalence of Cognitive and Mood-Related Symptoms in a Large Cohort of Perimenopausal and Menopausal Women

**DOI:** 10.1192/bjo.2024.497

**Published:** 2024-08-01

**Authors:** Dan Reisel, Clair Crockett, Sarah Glynne, Aini Kamal, Louise Newson

**Affiliations:** Newson Health, Stratford-upon-Avon, United Kingdom

## Abstract

**Aims:**

Current NICE guidance (NG23) lists hot flushes and night sweats as the most common symptoms associated with the perimenopause and menopause. Consequently, many clinicians, and the public in general, often associate menopause primarily with vasomotor symptoms. However, psychological symptoms are also common in the perimenopause and menopause. Failure to recognise the link between menopause and mental ill-health means that many women are unable to access the support and treatment they need; women are often prescribed antidepressants and anxiolytics, but hormone replacement therapy (HRT) is more effective for symptoms rooted in hormone deficiency. The aim of this survey was to assess the prevalence of negative mood symptoms in peri- and post-menopausal women, and the response of mood symptoms to HRT.

**Methods:**

We administered a modified version of the Greene Climacteric Symptom Questionnaire (Greene 1976) to all new patients attending the Newson Health Menopause and Wellbeing Clinic in Stratford-upon-Avon, between 1 November 2022 and 30 June 2023. Patients initiated on HRT were followed-up after 3 months and asked to complete the Symptom Questionnaire again. Data were collected from electronic health records and analysed using descriptive statistics.

**Results:**

978 women were included in the study. All patients were started on HRT. A third of patients (32%) of patients, were also started on transdermal testosterone. None of the patients discontinued their treatment during the study period. The five most prevalent symptoms were: feeling tired or lacking in energy (96%); memory problems (93%); difficulty in concentrating (91%); irritability (90%); and feeling tense or nervous (90%). Hot flushes and night sweats were much less prominent in this cohort, ranked at 18th and 14th place respectively. All symptoms improved after treatment with HRT +/- testosterone for 3 months. Overall, ‘profound low mood’ (loss of interest in all things) improved the most (69% improvement in symptom scores), followed by ‘attacks of anxiety and panic’ (61% improvement in symptom scores).

**Conclusion:**

Understanding and recognising the common symptoms that women are likely to experience in the perimenopause and menopause is vital to reduce barriers to appropriate care. This study suggests that cognitive and mood-related symptoms are highly prevalent and may be more common than hot flushes and night sweats. For most women, these symptoms improved after a short course of HRT. Longer follow-up is needed to assess any additional response to HRT given for longer periods, after individualisation and optimisation of the dose and regimen.

